# Fingerprinting materials oxidation using laser-induced XUV spectroscopy (LIXS)

**DOI:** 10.1007/s00216-025-06067-9

**Published:** 2025-08-20

**Authors:** Davide Bleiner, Sharath Rameshbabu, Janosch Von Ballmoos

**Affiliations:** 1 University of Zürich, Winterthurerstrasse 190, 8057 Zürich, Switzerland; 2 Swiss Federal Laboratories for Materials Science & Technology, Überlandstrasse 129, 8600 Dübendorf, CH Switzerland

**Keywords:** LIXS, Oxidation, Plasma, Laser, Spectroscopy, Manganese, Battery

## Abstract

Laser-induced XUV spectroscopy (LIXS) is an emerging plasma-based microanalytical technique that offers access to the early, “background-uncontaminated” (pristine) stages of laser-induced plasma evolution. In this study, the capability of LIXS to resolve not only the elemental composition but also the oxidation state of complex materials is investigated, i.e., by exploiting radiative recombination dynamics. Using a spatial-filtered detection scheme, the evolution of plasma emission was tracked, revealing characteristic spectral features associated with electron recombination into unoccupied s or d atomic orbitals. The latter retain a distinct fingerprint of the parent material’s oxidation state. Preliminary experiments on LiF, Al, and Ni served to calibrate the relationship between expansion kinetics, emission time delay, and atomic structure. Reference samples, including lithium manganese oxides with well-defined stoichiometries, were used to correlate LIXS spectral fingerprints to manganese oxidation levels. The results indicate that, contrary to the observations for the thermalized LIBS plasma, the LIXS laser plasma does not erase information on the material’s stoichiometry. Instead, the recombination features in the XUV region directly reflect the electronic configuration of the parent material. This work introduces LIXS as a novel route for rapid, stoichiometry-sensitive mapping of oxidation states, with immediate applications in the quality control of energy materials and battery components. The approach lays the foundation for the development of oxidation-specific plasma spectroscopy, extending the analytical power of laser ablation far beyond elemental analysis.

**Graphical abstract:**

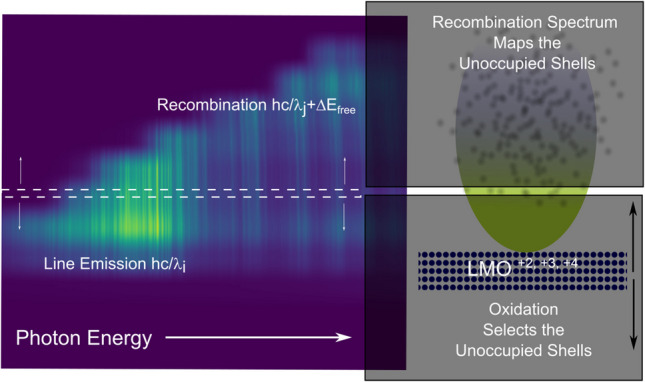

## Introduction

Laser ablation microanalysis has revolutionized how we map solid materials, offering a rapid and direct method for spatially resolved surface composition. Among these techniques, laser-induced breakdown spectroscopy (LIBS) has earned a prominent place thanks to its versatility, minimal sample preparation, and suitability for field use. LIBS can detect nearly every element, including light elements such as hydrogen [1] that are often difficult to analyze by contending methods. The basic assumption is that the laser-produced plasma spectrum mirrors the elemental composition of the irradiated materials. LIBS’ ability to operate in ambient conditions with compact, portable systems has opened doors across disciplines from planetary exploration [2, 3] and environmental monitoring [4, 5] to biomedical diagnostics [6, 7] and industrial quality control [8, 9].

Despite these advantages, LIBS faces a fundamental challenge: the precision of its measurements is limited by the large flicker noise of the laser-induced plasma expansion [10]. Thereafter, the heterogeneity of a material cannot be quantified better than the LIBS repeatability. To mitigate this drawback, laser-induced XUV spectroscopy (LIXS) has emerged as a “Columbus egg” smart approach. In fact, by means of shifting the detection window into the extreme ultraviolet (XUV) range, emitted when the plasma is still very hot, dense, and not yet ballistic, LIXS opens a view into the non-thermalized spectrum (non-LTE). Unlike LIBS, which must contend with continuum background and reabsorption effects, LIXS benefits from a background-free spectral region and strong line emissions from light and heavy elements alike [10–13], more closely mirroring the parent materials. The term “*pristine plasma*” has been introduced to indicate that the LIXS plasma is not yet contaminated with unspecific background.

What makes LIXS particularly worth studying is its sensitivity to fast spectrochemical processes, such as radiative recombination, which can occur within nanoseconds after plasma ignition, i.e., within the duration of the laser pulse. This offers a new opportunity to study transient plasma phenomena and potentially extract chemical information from the signal structure. The ability to extract sample-specific features that go beyond the elemental composition is important, e.g., in the case of specific stoichiometries that are mirroring the oxidation of the analyzed material.

In this study, the spatial-filtered emission of a LIXS plasma was studied to investigate the spectral features in detail. By slicing the plasma evolution, one aims to better understand how key processes such as radiative recombination and line emission unfold and whether LIXS can encode chemical information. A detailed analysis of the spectral features from a large selection of target materials revealed that the LIXS spectrum strongly depends on the material chemistry. The assumption that the plasma erases the memory of the material oxidation because it thermalizes its ion population, as known in LIBS, cannot be supported a priori in LIXS and needs investigation. This opens new possibilities for the fast mapping of energy materials with accurate inspection of the local redox characteristics.

## Experimental

### Samples

The samples used in this study consisted of a selection of materials. First, a 2-in. lithium fluoride (LiF) optical plate with a thickness of 4 mm was procured from Golem IMS GmbH. Composed of lithium (Li) and fluorine (F), LiF is a well-established optical material, renowned for its exceptionally low refractive index in the infrared region and its high transmittance in the ultraviolet (UV) spectral range. The exceptional high homogeneity is useful to test signal-to-signal fluctuations. In addition to its optical properties, LiF is gaining attention as a promising component in lithium-ion battery technologies, particularly as a stable salt for electrolytes [14]. LiF possesses a large electronic bandgap of 13.6 eV, which significantly hinders single-photon ablation processes. The electronic structure of lithium, with only three electrons, limits the number of available transitions for both neutral and ionized states when compared to heavier elements. Notably, Li^2^⁺ (Li III) exhibits a strong emission line at a wavelength of 13.5 nm, which is of particular interest for applications in the extreme ultraviolet (XUV) region. Given these characteristics, LiF was selected as a reference material for wavelength calibration and as a starting point for the spectroscopic investigations carried out in this study.

To establish a calibration function for lithium concentration based on XUV emission signals, four calibration standards were prepared using stoichiometric mixtures of lithium oxide (Li_2_O) and various manganese oxides, specifically (manganosite) MnO, (pyrolusite) MnO_2_, and (hausmannite) Mn_3_O_4_. These mixtures were thoroughly homogenized and pressed into pellets to ensure consistent analytical performance. The prepared samples are designated as *mng*, *hsm25*, *hsm71*, and *pys*, containing varying proportions of Li_2_O and Mn_x_O_y_, as detailed in Table 1. These samples are globally referred to as LMO.

**Table 1:** LMO sample materials: composition and determined values from the LIXS spectral integration (see data in Fig. 8)

Formula	Mn^II^O	Mn^II^Mn^III^_2_O_4_	Mn^II^Mn^III^_2_O_4_	Mn^IV^O_2_
**Phase**	**Mng**	**Hsm25**	**Hsm71**	**Pys**
Mn^+2^	70.60%	22.70%	20.40%	
Mn^+3^		45.40%	40.70%	
Mn^+4^				59.00%
Li (tot)	4.10%	2.50%	7.10%	3.10%
O(tot)	25.3%	29.3%	31.9%	37.9%
O (stoichiom. from Mn)	20.6%	26.4%	23.7%	34.4%
O (stoichiom. from Li)	4.7%	2.9%	8.2%	3.6%
**Total**	**99.99%**	**99.92%**	**100.11%**	**100.04%**
d-series rel. integral	0.0%	0.8%	23.3%	8.1%
s-series rel. integral	100.0%	99.2%	76.7%	91.9%
Determined total charge	0.9	0.4	0.4	4.0
Nominal charge	+ 2	+ 2.7	+ 2.7	+ 4

Finally, pure Al and Ni metallic standards in the form of 1-in.-diameter pellets with a thickness of 4 mm were used for elemental mapping calibration. These served as reference samples to validate the elemental distribution and signal calibration during the mapping process.

#### Laser ablation experiments

##### Spatial filtering setup

Details are reproduced from previous publications [10–13]. A Q-switched Nd:YAG laser (λ = 532 nm, *τ* = 6 ns) was imaged (*f* = 300 mm) onto the sample surface to a spot size of approximately 100 µm. The focusing was carried out by imaging, and an auto-focus made sure that the fluence of irradiation was constant within the 1% pulse-to-pulse fluctuation of the laser. The imaging approach guarantees homogeneous fluence at all settings within the depth of focus of approx. 1.9 mm. The experiments were conducted in a vacuum chamber maintained at a base pressure of 10⁻⁶ mbar to minimize XUV absorption by ambient gases. A single laser pulse, delivering 200 mJ of energy, was used for each measurement. The resulting plasma emission was collected through a 50-µm entrance slit and analyzed using a custom-built XUV spectrograph, as described in Qu et al. [12]. The emitted radiation was diffracted by a variable line spacing (VLS) grating positioned at a grazing incidence angle of 3°. The spectrally dispersed light was then imaged onto a back-illuminated CCD camera, cooled to –60 °C to reduce thermal noise. The spectrograph provided coverage in the XUV range from 4 to 20 nm. By changing the depth of the sample mount stem, one would recede or advance the virtual source for the XUV spectral collection. In fact, by moving backward the sample mount, the plasma would have to expand over a proportionally longer height to transit in front of the viewing window. Thereafter, spatial filtering of the observation height of the plasma would give a spectral signature at different expansion times. As the expansion is in vacuum, the height/time calibration (plasma expansion speed) is assumed to be constant.

**Fig. 1:**
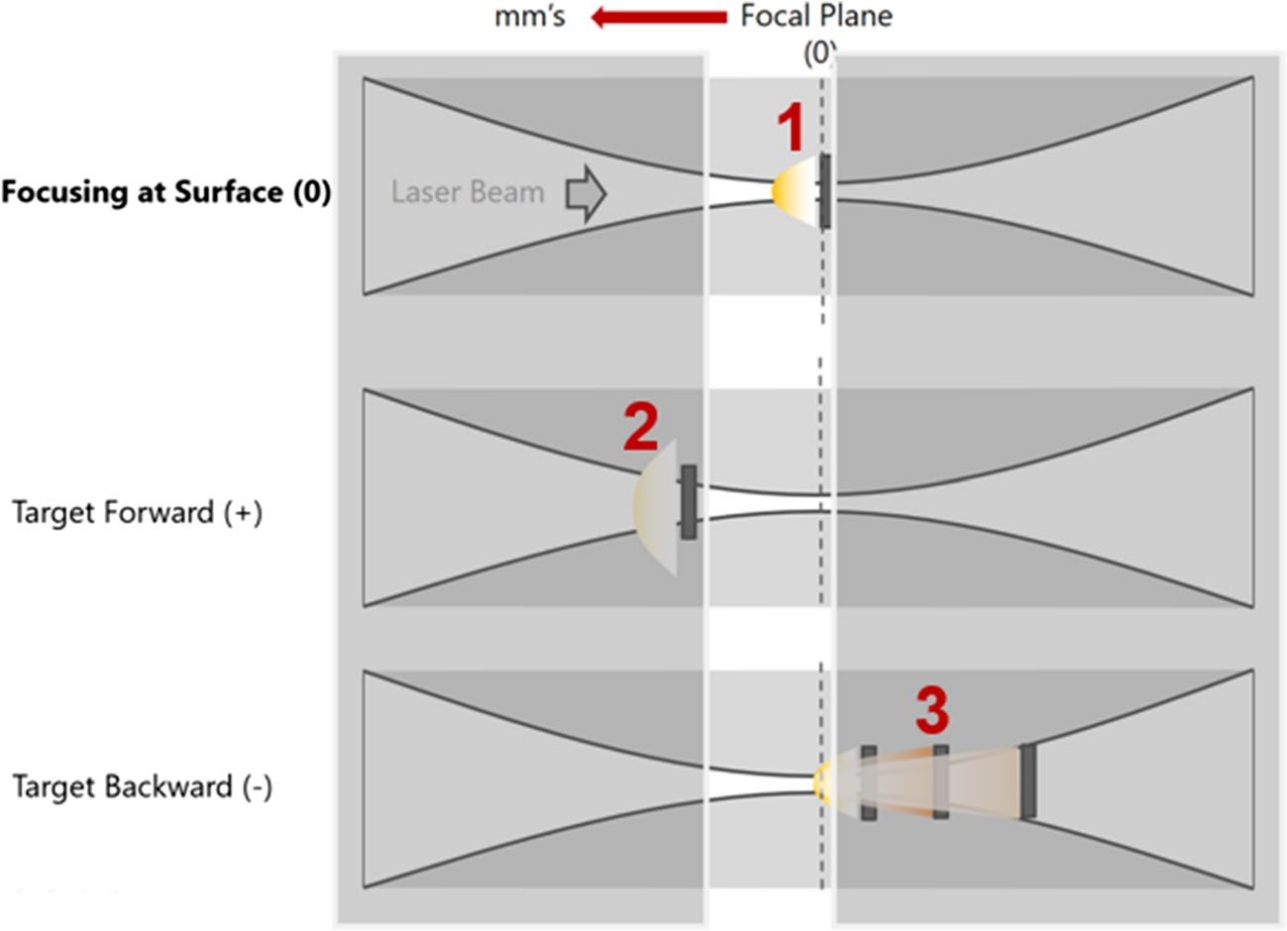
Schematic of the spatial filtering experiments. The laser is imaged on the target to generate a plasma. Its XUV emission is collected within the spatial window of the slit width. In the top panel (pos. 1), the target is right at the back plane. In the middle panel, the target is at the front end (pos. 2), which prevents any emission observation. In the bottom panel (pos. 3), the target is hidden behind the aperture and the emission is observed only after a short delay, when the plasma emerges within the slit width. The numbered positions are indicated in the spectral mapping of Fig. 5

Figure 1 summarizes the setup schematically, showing three conditions. In the top panel (pos. 1), the target material is positioned aligned with the spectrometer slit back end. The slit width is 50 mm. Therefore, one is able to observe a signal integrated over the first 50 mm of the plasma height above the sample. In the middle panel (pos. 2), the target is advanced beyond the forward edge of the slit. With that, obviously no signal should be collected, which is confirmed by the data as discussed in the “Role of observation height” section. In the bottom panel, the target is positioned at different distances beyond the backward edge of the slit (pos. 3). Thereafter, a signal is collected only after the plasma has expanded to emerge within the slit width. The integration height is still fixed to the slit width of 50 μm. However, the delay, given by the spatial filtering that the slit knife edge exerts, permits collecting the signal after a certain time lapse of the plasma expansion, to isolate specific short-lived processes. With that, one was able to collect simultaneous full spectra at specific time delays. These data are important to track the evolution of the spectral signal, and with that the emitting species in the aging plasma. Thanks to a modelling of the expansion distance, one is able to calibrate the time of expansion. This is discussed in the following section.

##### NELIXS experiments

A dispersion of gold nanoparticles was utilized to study the signal enhancement reported for LIBS [15–17] (called NELIBS) also in LIXS (nanoparticle-enhanced LIXS or NELIXS). Specific size classes were studied with 10, 50, 100, 150, and 200 nm. The size did not show any difference in the enhancement. The dispersion from Aldrich was in a citrate buffer. The dispersion prepared with 0.5 μL Au nanoparticle solution was pasted on the sample surface homogeneously. Care was taken to analyze the fresh sample (i.e., within 10–15 min) to prevent evaporation, which showed to impact dramatically the precision of the results. The enhancement was compared to the literature. The target was aluminum. X-ray fluorescence analysis of the target confirmed the purity with minor contamination of Mg and Cu. To minimize surface dirt, the sample was always pre-washed with ethanol.

#### Space-time calibration of the laser plume expansion

When a high-intensity laser irradiates a solid target, a plasma forms and expands rapidly into the surrounding environment (in the case of LIXS at a few mbar, considering the vapor plume outgassing). The expansion dynamics can be described by the Sedov-Taylor *blast wave* model [18–20], originally developed for explosions of warfare, assuming that: (i) the energy release is instantaneous, (ii) the expansion is one-dimensional or spherically symmetric (can be adapted for cylindrical geometry), (iii) no significant losses are considered (adiabatic process) so that the conversion into mechanical energy is tracked, (iv) the ambient gas behaves close to an ideal gas.

Several experimental studies have confirmed that the Sedov-Taylor blast wave model remains valid well beyond hundreds of ns [21], extending into the microsecond regime [22, 23], including under low-pressure conditions. While minor deviations may arise due to radiative losses [24], ionization, and multiphase effects, the model continues to provide a reliable scaling framework as long as expansion inertia dominates. Observations consistently show that plume dynamics follow power-law behavior, with hydrodynamic behavior persisting until LIBS stages (μs´s) where diffusive or ballistic regimes shall emerge.

A modelling was carried out to obtain analytical calibration curves of the experimental plume position versus the corresponding time of plume age. The key modelling parameters are the explosion/ablation energy *E*, the ambient density *ρ*_0_, the adiabatic index *γ* = 5/3 (for monoatomic gases), the time since explosion *t*, and the geometric factor *α*≈1 (depends on dimensionality). The blast wave radius (or plume expansion distance *R*) at the shock-front is given by

1 $$R\left(t\right)=\alpha {\left(\frac{E}{{\rho }_{o}}\right)}^{1/5}{t}^{2/5}$$

The velocity is easily obtained by differentiation, as follows:

2 $$V\left(t\right)=\frac{dR}{dt}=\frac{2R}{5t}$$

while the acceleration can be computed numerically. The Sedov-Taylor equations were implemented in Python using NumPy and Matplotlib. All simulations used a time grid defined as follows: full time range plots: *t* = [0.001,10] μs with 500 points; high-resolution early-time plots: *t* = [0.001,0.1] μs with 1000 points (adaptative mesh to capture the faster expansion scale). The acceleration was computed via numerical differentiation of the velocity vector using *numpy.gradient*. All results were exported as CSV files for further analysis and visualization in SciDavis software.

To account for the effect of the material, the initial energy deposition is corrected for the absorptivity and reflectivity at the operating laser wavelength. For the case investigated here, the following considerations were made:

- LiF: Transparent at many wavelengths → low absorption → weak plasma unless multiphoton or avalanche ionization occurs → likely low *ρ*_0_, fast expansion, but weak plasma
- Al: High reflectivity (~ 90%) in 532 nm → lower effective energy coupling unless treated/coated → intermediate *ρ*_0_, depends on laser absorption and fluence
- Ni: Higher absorption than Al in UV and XUV → stronger initial plasma at shorter wavelengths → denser plasma, higher *ρ*_0_, slower but more energetic

Therefore, these three materials are an excellent benchmark to study a wide range of cases with increasing strengths. In fact, the Sedov-Taylor theory assumes that the plasma expands into an ambient gas of density *ρ*_0_, but in a laser-produced plasma, the “ambient” medium just outside the target is the background gas + vapor plume from the material itself. The vapor density immediately after ablation can vary drastically depending on the material’s bulk density, atomic weight, and enthalpy of vaporization. Table 2 summarizes the values utilized for the present modelling.

**Table 2:** Values utilized for the present modelling

Material	Bulk density (g/cm^3^)	Molar mass (g/mol)	Vaporization temp (°C)
LiF	2.64	25.94	1670
Al	2.70	26.98	2519
Ni	8.90	58.69	2732

The laser pulse used in this study had the following parameters, corresponding to the experimental conditions discussed above: wavelength λ = 532 nm; pulse duration (FWHM), 6 ns; pulse energy, 200 mJ; focal spot diameter, 100 µm. To account for realistic energy coupling to the plasma, an energy absorption efficiency of 30% was assumed, resulting in an effective explosion energy of 60 mJ.

## Results and discussion

The aim of this study was to investigate in detail the features of the LIXS spectrum to relate it to fingerprinting the sample chemistry. As a first attempt, one had to make sure that the spectral structure is consistent through the plasma observation height above the sample surface. The study identified a certain dependence as a function of plasma observation height. For consistent alignment of the viewing height, the results were, however, very reproducible within less than a percent, as limited only by the laser pulse to pulse stability. It was possible to interpret the specific features as related to the dynamics of the plume expansion, with an optimum zone in specific viewing heights.

Next, the availability of free electrons was studied, as being one important factor affecting the bound–bound transition and in particular the electronic recombination. Finally, it is shown that the plasma preserves a memory of the sample material stoichiometry, since different material stoichiometries gave correlated spectra, well beyond the bare elemental composition. This is an important conclusion, which opens the possibility to fingerprint the spatially resolved (mapping) material oxidation.

### Role of observation height

**Fig. 2:**
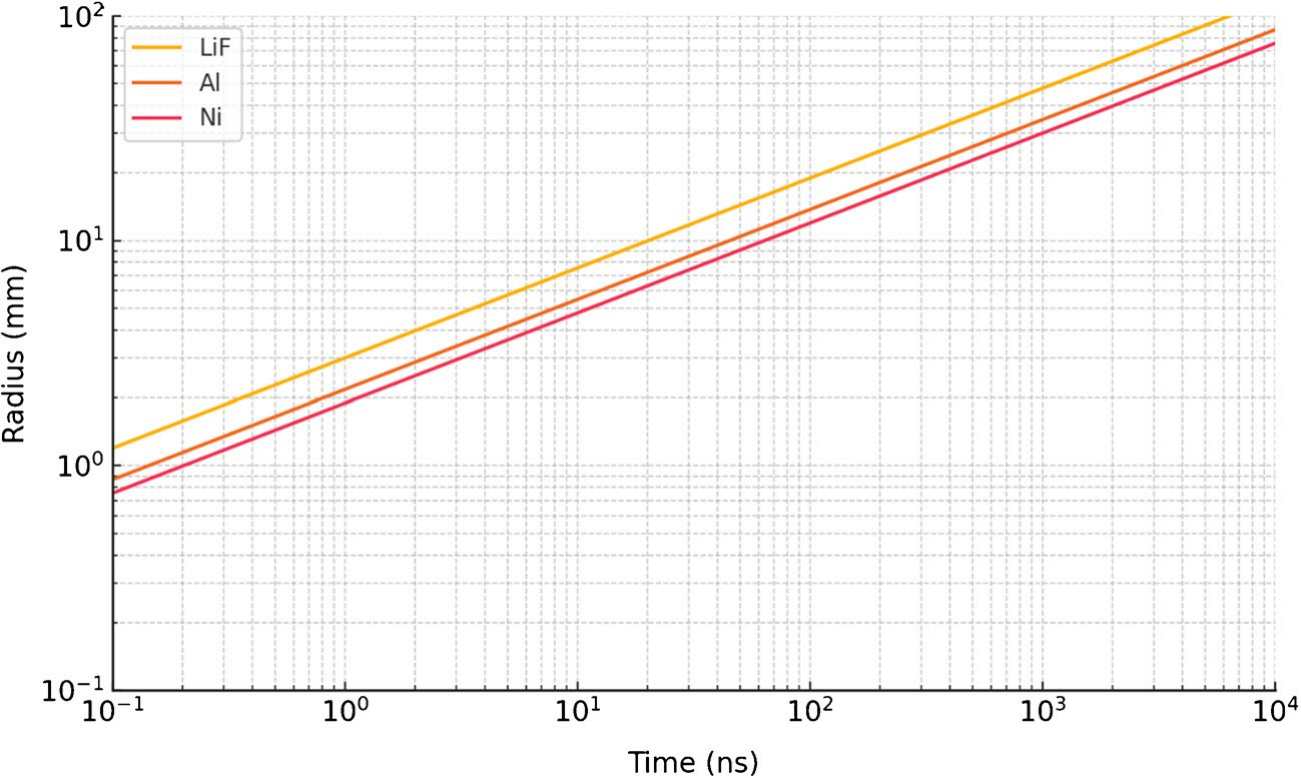
Radial distance travelled by the plasma plume as a function of time for three materials, at 10^–3^ mbar ambient (see text for discussion)

**Fig. 3:**
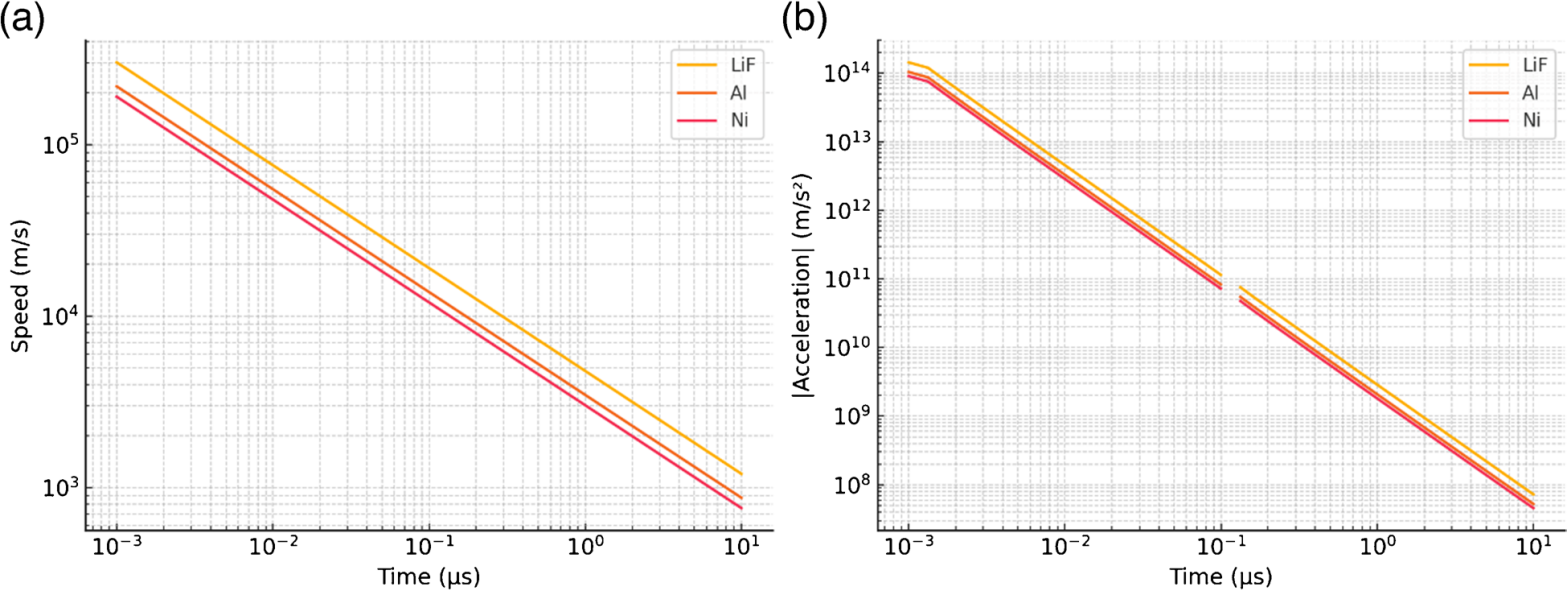
Kinematic characteristic of the plume expansion at 1 mbar ambient. **a** Speed, **b** acceleration (see text for discussion)

Figure 2 shows a computational calibration (based on the blast wave model discussed above) of the plume expansion position versus time, for laser-produced plasmas from LiF, Al, and Ni targets. The slowing down of the expansion is to be minded in the logarithmic scale. The plot serves as a semi-quantitative calibration between position and time of the laser-plume expansion front. Figure 3 shows the calculation details of the speed and acceleration for the three target materials, with log–log scale. One notes the higher speeds (and acceleration) for the LiF plume as compared to the Al and Ni cases. The speed is hypersonic (i.e., greater than fivefold the speed of sound) as long as a few microseconds, which supports molecular dissociation and ionization, the occurrence of a shock layer, and a decrease in volume behind that layer for energy conservation, as typical for the hypersonic regime [25]. The rate of speed change (deceleration) is essentially geometric as visualized in the logarithmic scale. Then the flow transitions to a supersonic/transonic regime (0.8 to 1.2 Mach) that condition does not support the replenishment of plasma state. In fact, in the hypersonic regime, ionization is self-sustained.

**Fig. 4:**
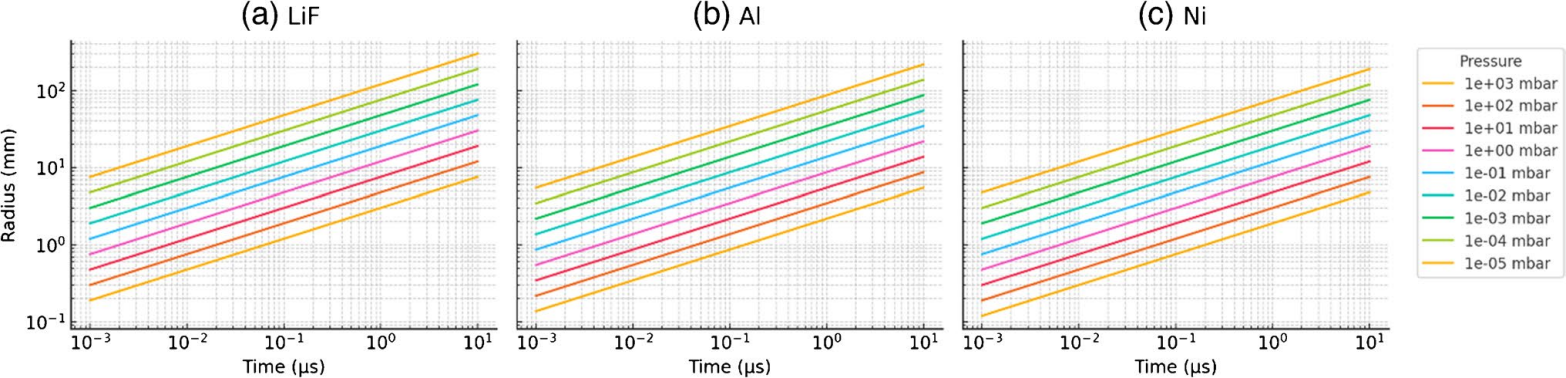
**a**–**c** Plume expansion distance as a function of time, in different ambient pressures, for the three materials (see text for discussion)

One can question how much the ambient pressure impacts on the laser plasma dynamics. This point is important to understand the substantial difference between LIXS (operated at low-pressure ambient) and classical LIBS (which is operated at atmospheric pressure) as well as understanding the impact of the plasma plume debris. Figure 4 summarizes the distance-time calibration curves (log–log scale) for the three materials, as a function of ambient pressure, from atmospheric down to high vacuum. One observes that the range is as high as approx. 10 mm, and this can modulate the position of the plume front. Therefore, for the present experiment, a good vacuum buffer with a high capacity turbopump was used to stabilize the expansion fluctuation. Obviously, expansion is faster at lower ambient pressure, while for a given background pressure, LiF expands faster, followed by Al, then Ni, due to vapor density scaling.

**Fig. 5:**
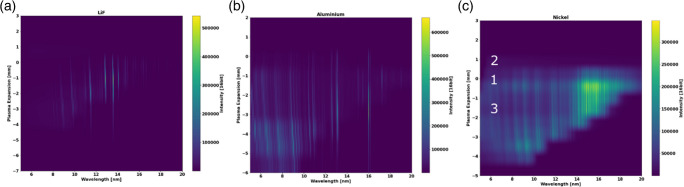
Heatmap of the LIXS spectra structure at different time delays (or plasma expansion height) for **a** LiF, **b** aluminum, and **c** nickel (see text for discussion of feature). The positions 1, 2, and 3 refer to Fig. 1

Figure 5 maps the collected spectra at different plasma expansion heights for the three target materials. Figure 5a shows the LiF spectrum. The limited number of bound–bound lines is easily explained: Li has a simple shell with limited XUV transitions. Fluorine is an anion with almost filled shells, such that besides limited transitions, it is a repeller for free electron recombination. The *λ* = 13.5 nm Li line is well documented also for its interest for the semiconductor industry as a candidate EUV lithography source [12, 26–28]. Besides this line, Li has an *λ* = 16.8 nm line. All Li lines tend to maximize at approx. 1 mm plasma expansion height above the target sample. From the calibration position-time discussed in the modelling section (section “Space–time calibration of the laser plume expansion”), this position corresponds to approx. 0.1 ns. Therefore, it happens at the leading edge of the laser pulse. This may suggest an interaction and re-ignition of the emission; i.e., the laser pulse feeds the plasma compensating the radiative energy losses. In fact, from all results obtained, this region looks to be the optimum position, with the most intense emission, for all the observed lines. This is clearly associated with the effect of the laser pulse. However, the other lines shown in Fig. 5a, attributed to fluorine, do extend their lifetime to plasma expansion heights as long as 4 mm (approx. 2 ns). It should be noted that the lines in the spectral region *λ* < 12 nm tend to initiate after 1 mm (approx. few hundreds of ps), which may be due to the preference for cooler plasma conditions as soon as the rising of the laser pulse has passed the peak.

Figure 5b shows the emission of aluminum. The more complex atomic structure as compared to Li explains the richer set of transitions observed. Interestingly, the region *λ* < 12 nm is characterized by a group of lines, which were previously explained as due to radiative recombination [13]. The lines have a secondary optimum at approx. 4 to 5 mm. These viewing heights correspond to approx. *τ* = 3.5 ns, which is in the trailing edge of the laser pulse. Therefore, the extinction of the pulse leaves an undressed plasma which acts as a rapid recombination sink, suddenly lacking the feed of laser energy. This would indicate that radiative recombination lines in LIXS, as discussed in Rameshbabu et al. [13], would prefer a larger plasma expansion height. This phenomenon was observed frequently in the lab, especially in combination with the analysis of precursors of energy materials, with elements such as Cr, Mn, Co, and Ni, often with different oxidation states (see discussion below).

It should be noted that LIXS, differently from LIBS, is a closed system. In fact, LIBS is typically carried out in atmospheric air and thus the chemistry of the plasma plume is affected by the entrainment of ambient N_2_, O_2_, etc. In LIXS, the plasma expands in a low-pressure environment in order to mitigate the attenuation of XUV. Thus, the parent material chemistry is preserved.

Figure 5c shows exactly the Ni spectra at different plasma expansion heights. The tag numbers indicate the regions, as given in Fig. 1. In pos. 1, one observes the optimum, which confirms the discussion made above associated with the laser pulse reheating. In pos. 2, the signal drops to zero because the target is past the front edge of the knife edge window, where the light is fully blocked off. This result is experimentally important to demonstrate that the level of stray light is negligible, and all regions are space-integrated exclusively to the emission within the slit width. Figure 5c also confirms the “radiative recombination” region at large plasma expansion height (approx. 3.5 mm or 4 ns). In agreement with the expansion effect of the atomic mass, as discussed in the modelling “Space–time calibration of the laser plume expansion” section, this region shifts its spatial position because it is associated with the same time delay of the laser pulse extinction. Comparing with Fig. 2, one concludes that a time delay of approx. 4 ns occurs at the following plasma expansion height for the various materials: 5.1 mm for LiF, 3.8 mm for Al, and 3.2 mm for Ni. The onset range is not exactly like the FWHM of the pulse (6 ns) possibly because the laser pulse is asymmetric (skewed Gaussian), with a steep leading edge and a tailed trailing edge. The matching is however quite close within a few percent. The fact that the spectral lifetime tends to reduce at longer wavelengths (lower transition energy) could be due either to (i) a stricter dependence on the pulse duration or (ii) a shift of the spectral emission during the plasma expansion. Also, a combination of both aspects may be true.

It is noteworthy to discuss the spectral line tailing shape, as a consequence of the mechanism of emission. Negative tailing (i.e., towards shorter wavelengths) is attributed to radiative recombination. The capturing of free electrons to an unoccupied level provides emission that is the sum of two terms: (i) the binding energy of the unoccupied level involved (edge-bound), plus (ii) an additional continuum due to the kinetic energy of the electron (free-bound). As the kinetic energy follows a (Maxwellian) distribution, the line shape is modulated accordingly. This means also that for shorter wavelengths (i.e., closer to the edge), the tailing is less pronounced. The occurrence of such negative tailing is indicative of fast plasma cooling, typical of the corona after the plasma has expanded over the Debye length [29].

On the other hand, the positive tailing (towards longer wavelengths) was observed, too. This is attributed to electron-photon scattering [30], with a partial loss of energy. Regions with higher plasma density and opacity offer a higher cross-section for such phenomena. In this respect, this is associated with complementary conditions as in the case of the negative tailing. This permits associating the emission with particular stages of the plasma expansion as well as regions.

This study has shown that the optimization of the observation height is important to enhance the signal-to-background ratio. As discussed, “continuum” could be due to the radiative recombination that occurs close to the line emission and slightly higher energies. In cases where the radiative recombination signal should be enhanced, as discussed below, one should look for a larger plasma expansion height of LIXS observation.

### Role of free electron availability

In the analysis of chemical fingerprint, one has to make an important distinction between the behavior of the line spectrum (bound–bound transitions) and the radiative recombination spectrum (free-bound transitions). The spectral structure is determined by the atomic physics of the analytes, but also by the availability of free electrons. The latter is influenced by the ionization of the plasma, as an effect of the laser pulse energy as well as its chemistry. Structural characteristics of the sample materials are also important. Several authors have shown a substantial LIBS signal enhancement [15–17, 31–33], especially for metallic samples, when the surface is prepared with a coating of metallic nanoparticles (so-called NELIBS). The very reproducible observation has been interpreted with a number of processes, ranging from surface plasmonic effects to enhanced availability of free electrons in the plasma. As the ablation per pulse (ablation yield) was not observed to increase, one would presume that the reason for the NELIBS signal enhancement is pragmatically to be attributed to processes in the plasma. The presence of metallic nanoparticles does scale the availability of free electrons in the plasma.

**Fig. 6:**
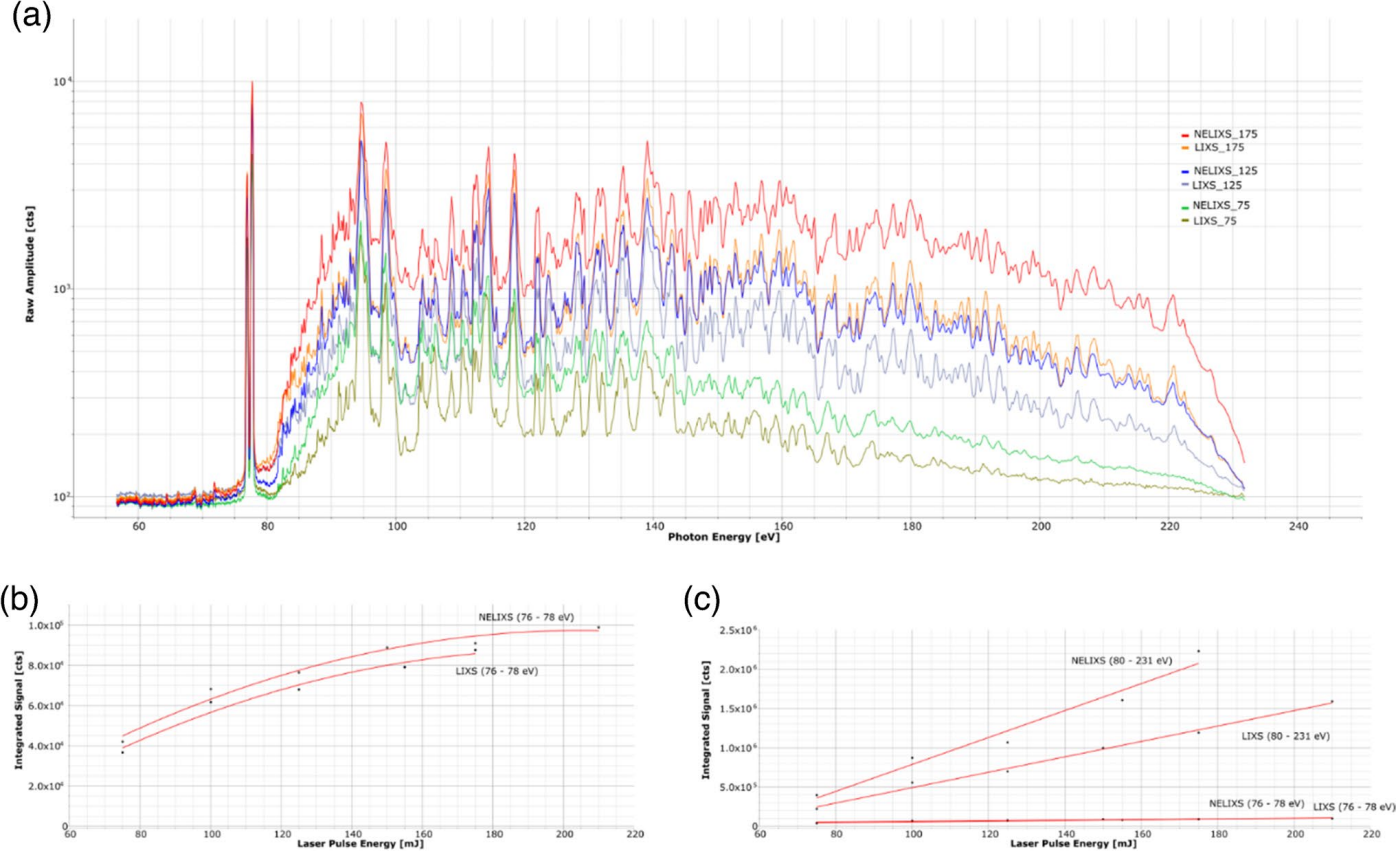
LIXS spectral signal from aluminum, with and without preparation of the surface with a coating of nanoparticles. **a** Comparison of the LIXS and NELIXS spectra at three different laser pulse energies, i.e., 75, 125, and 175 mJ. **b** Integrated intensity of the Al lines at 77 eV as a function of laser pulse energy, showing a saturation. **c** Integrated intensity of the radiative recombination continuum (80–231 eV)

In order to investigate all these discussed effects, specifically for the case of LIXS, the XUV response of an irradiated aluminum target, with and without nanoparticles, was studied. Figure 6a summarizes results obtained irradiating Al with (NELIXS) and without (LIXS) nanoparticles coating. Panel a shows the raw signal without background correction, for three laser pulse energies (25, 75, and 175 mJ). One notes the characteristic Al line at approx. 77 eV, and the large radiative recombination band in the 80–231 eV range. The application of nanoparticles produces a remarkable enhancement, such that for instance the NELIXS at 125 mJ is comparable to the 175 mJ LIXS. Henceforth, the application of nanoparticles produced effects that correspond to approx. 50 mJ additional energy. This dependency is non-linear. In fact, at lower energy, the 125 mJ LIXS is still higher than the 75 mJ NELIXS (also 50 mJ difference).

Figure 6b and c show integrated signals for LIXS and NELIXS, as a function of laser pulse energy, after background subtraction. The background was taken off-peak at 60–70 eV and integrated for an equivalent integration range to the signal to correct. The plots show the trends in LIXS and NELIXS. The error bars in panels b and c are in the range of < 10^–1^ (i.e., 3–9%), so well smaller than the data point symbols (in fact the bars are plotted but not visible due to the tiny size). Figure 6b shows specifically the characteristic Al lines. The trend shows a negligible effect of the nanoparticle, while the laser pulse energy is the most important parameter. The trend is non-linear and tends to saturate at higher energy. Therefore, the operating energy is optimal to get the best signal. In Fig. 6c, the integrated signal is shown for both the characteristic lines (bottom curve) and for the radiative recombination in the region 80–231 eV, visible in Fig. 6a. One notes a factor of 2 enhancement from LIXS to NELIXS. These results suggest the enhancement of the free electron density, with more substantial collisional ionization and/or recombination. The control of the free electron number density is an important factor to determine the sensitivity to fingerprint the oxidation of the parent material. As the plasma maps the composition of the parent material, it is a standardized availability of free electron that can determine the radiative response. How much the sample material oxidation influenced the spectral fingerprint was investigated and discussed in the next section.

### Role of sample stoichiometry

The plasma is a “dynamic sample” amid between a gas and a metallic system. As a gas, an atom-centric description is accurate. With that, the definition of ionization, restricted to a single atom of the gas ensemble, implies the release of a valence electron. As a metallic system, an electron can be delocalized, with any of these three cases: (i) remains within a Debye length and represents a form of atomic oxidation, possibly recombining at a later time; (ii) is promoted to the conduction band, and it remains coupled with the plasma; (iii) is lost to the vacuum, if able to overcome its electrostatic pull to the rest of the plasma.

While any of these conditions can represent a specific moment in the laser plasma expansion dynamics, one must bear one important aspect in mind. The laser-induced plasma develops out of a solid target. The rapid sampling process, within less time than a laser pulse lifetime, does not suggest a full thermalization. The unsteady condition of the sampling, described by a non-LTE model, represents a form of transient quenching the solid. This guarantees a correlation between the parent solid and the emitting plasma in the LIXS regime. In fact, LIXS resorts on a transient plasma, while LIBS has thermalized and preserved the elemental composition.

**Fig. 7:**
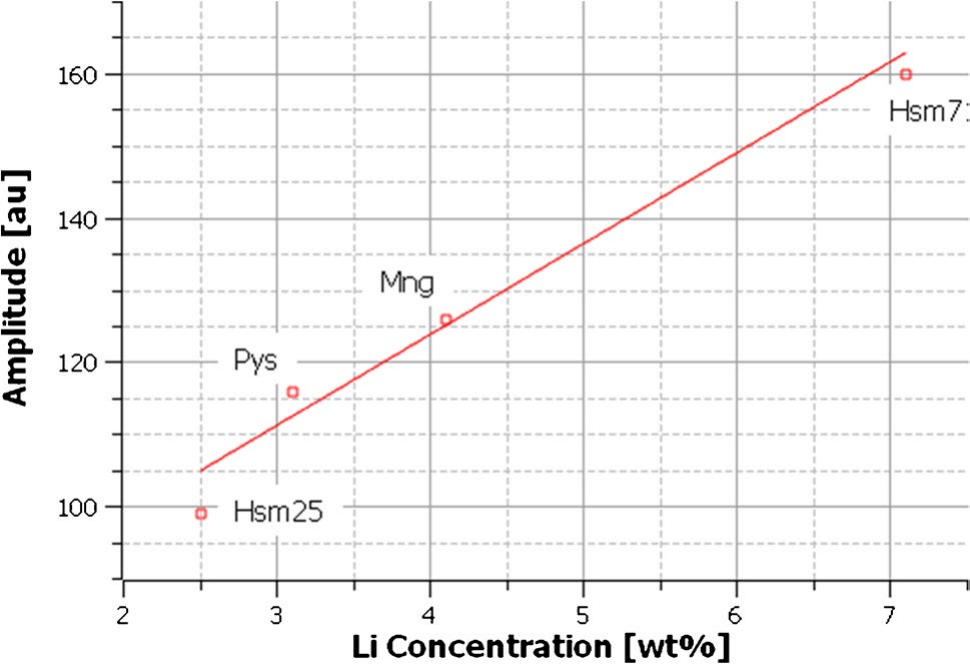
Signal response of the Li 13.5 nm line as a function of its concentration in the four manganese-bearing samples

LIXS spectra for Li_2_Mn_x_O_1+y_ (or LMO) [11] with different proportions of the Mn-phases manganosite (ox. + 2), hausmannite (ox. + 2, + 3), and pyrolusite (ox. + 4) were studied, as given in Table 1. The Li line (13.5 nm) signal was calibrated (see Fig. 7) with the amount of Li in the samples. Li is important as a functional element for battery materials. Here, it is also important as an electron donor, thus also influencing the free electron availability. The approach is an alternative to the NELIXS method (use of nanoparticle coating), as discussed in the previous section, in order to control the availability of free electrons. It was observed that the free electron activity determines the radiative recombination intensity and also the shells in which the electron recombines (see below).

**Fig. 8:**
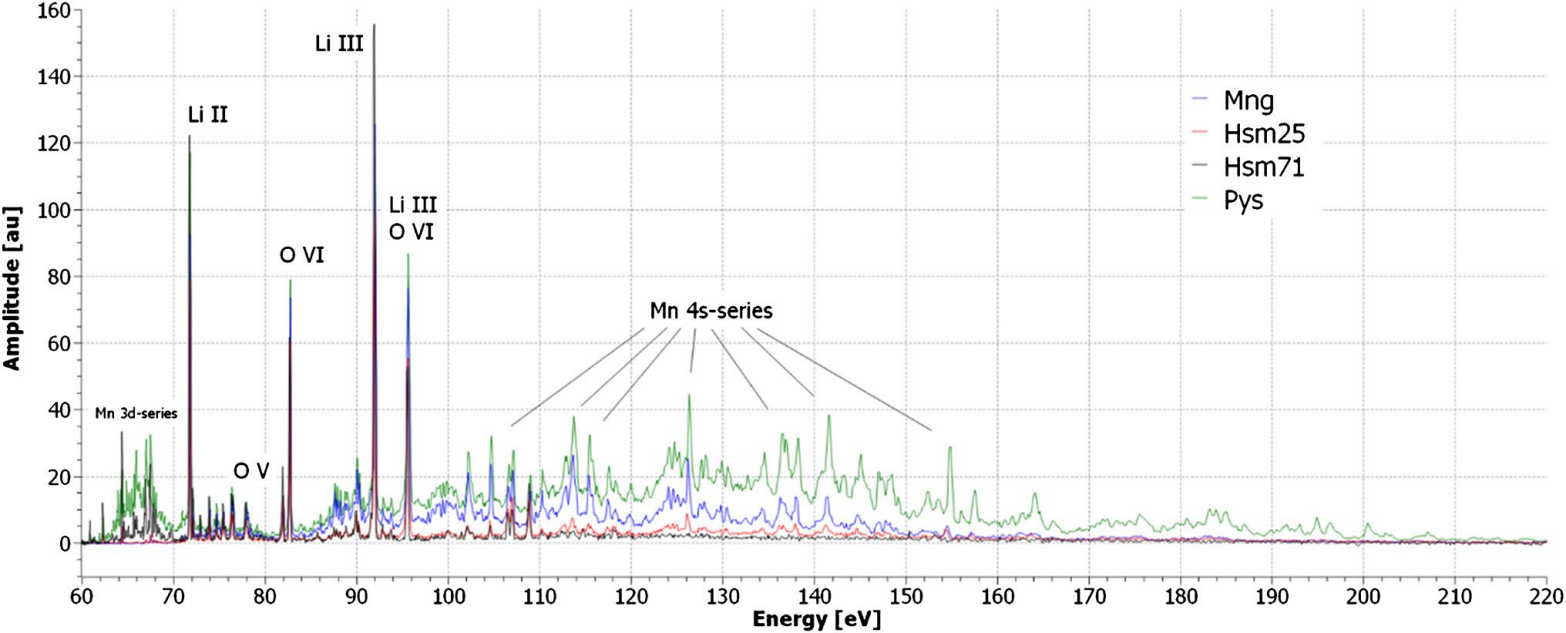
LIXS spectra of four LMO samples with different Mn-bearing phases, as given in Table 2. The radiative recombination to the canonical 4s shells and the 3d empty levels is visualized in Fig. 9 (see text for discussion)

**Fig. 9:**
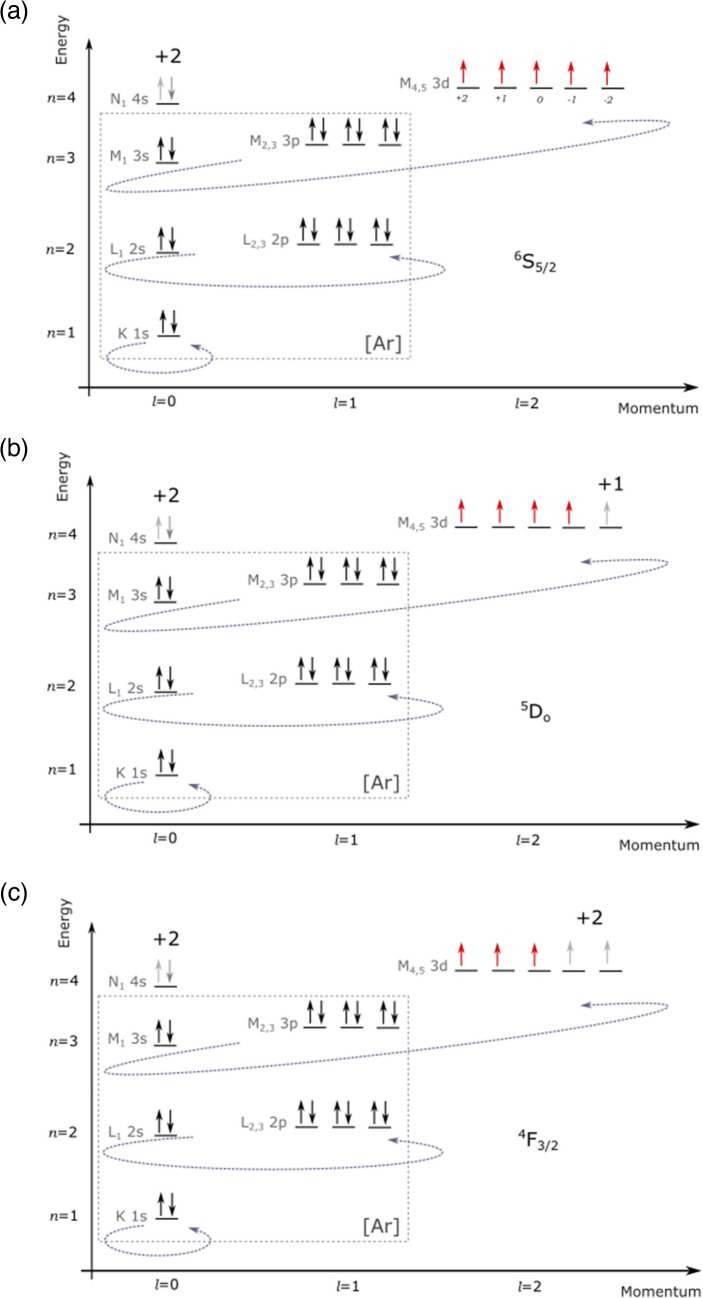
Grotrian diagram for the LMO phases of Mn. **a** + 2 as in pure Mng (or part of Hsm) shows a fully unoccupied 4s shell and half-filled (stable) 3d-shells. **b** + 3 as Hsm-component shows a mix of fully unoccupied 4s shell and one unoccupied 3d m_*l*_ =  − 2 shell (see m_*l*_ index below to the box). In Hsm, this component coexists with the + 2 shown in **a**. **c** + 4 as in pure Pyl shows a fully unoccupied 4s shell and two unoccupied 3d shells, such as m_*l*_ =  − 1, − 2. Levels not to energy scales. Filling only indicative but multiple configurations equivalent to these three cases are to be understood. Term symbols are given to visualize the spin–orbit coupling

In detail, Fig. 8 shows LIXS spectra for the four irradiated samples. One notes a clear structure that is qualitatively consistent across the various samples: a region with Li and/or O characteristic lines (approx. 70–80 eV), a region with 4 s radiative recombination electrons (approx. 80 to 220 eV), and a region with 3 d radiative recombination electrons (approx. 62—70 eV). Figure 9 summarizes the electron shell occupation for Mn with the three cases. Panel a shows the electron configuration for the + 2 case (as in pure manganosite), where the charge is induced for the loss of two 4 s electrons. Panel b shows the + 3 case, where besides the two 4 s electrons, also one 3 d electron is unoccupied. In hausmannite, the latter case coexists with the former case. Panel c shows the + 4 case (as in pure pyrolusite), which emerges from the 4 s and partly 3 d unoccupied shells.

Let us consider the distribution of Mn ion stages from a laser-irradiated LMO sample. If the ion stages are steps of the plasma thermalization process, as under a local thermic equilibrium (LTE), all stages in all samples should be populated. Their proportions are a function of the plasma temperature and independent of the original sample oxidation. As a matter of fact, instead, the spectra for the LMO samples irradiated are substantially different, even at comparable laser sampling and plasma temperature. The sample encodes information that is mapped in the recombination spectrum. A detailed atomic physics analysis suggested that the oxidation form and stoichiometry influenced the occurrence of certain spectral features.

To investigate this point, the s- and d-series (see Fig. 8) were integrated for the various LMO materials and normalized to the sum of these integrals in order to obtain the fractional proportion of the recombination for the various oxidation phases. These normalized integrals served as a proxy of the abundances of the electrons recombining to the s- or the d- unoccupied shells. Table 1 summarizes the results. While the manganosite (+ 2) and the pyrolusite (+ 4) are monophase, the hausmannite is a mixed phase with coexisting + 2 and + 3. Besides the compositions, Table 1 summarizes information derived from the integration of the spectra shown in Fig. 8. The extent of the mapping of the unoccupied shells must be put in relation to the availability of free electrons (see the “Role of free electron availability” section), besides the availability of unoccupied shells. These points are discussed in the following.

For the various LMO phases, the availability of unoccupied states is different, as shown in Fig. 9. Table 1 suggests that there is a strict correlation with the expected electronic population and unoccupied shells, following two simple criteria. First, half-filled shells are stable and are not receptive to a recombining electron. From the selection rules and the Pauli exclusion principle, half-filled shells are replenished only if fulfilling a condition of antiparallel spin. This is a very strict condition that lowers the probability of recombination to half-filled shells. Thereafter, the proportions of half-unoccupied d-shell filling are lower, as shown from the experimental integral summarized in Table 1. Fully unoccupied shells are thereafter mostly receptive to recombination. Fully empty shells are, however, only available in specific oxidation cases, as summarized in Fig. 9. This explains the trends observed in the LMO spectral signals and related integrals.

Second consideration, the integrals suggest that the s-shells are more receptive than d-shells, which is most likely due to symmetry considerations. In fact, it is observed that the s-series is the only receptive level for the manganosite. For the hausmannite and the pyrolusite, the s-series is substantially more receptive than the contending d-series.

As said, the availability of free electron density is important, ceteris paribus. This availability here can be either due to a larger ionization (e.g., + 4) or free electron population (NELIXS), or also due to the concurrence of higher Li abundance. Henceforth, for the hausmannite, the one with higher Li content was observed to have a larger proportion of d-shell population.

Finally, it was determined the total charge, from a weighted average of such percentages considering the fractions of + 2, + 3, and + 4 in the sample, as given in the top part of Table 1. Weighting the averages is very important to avoid misinterpretation of the experimental evidence. The approx. 20/40 proportion of + 2 and + 3 in hausmannite determined the fact that the obtained charge is not the simple average of 2 and 3 (which would erroneously imply a 50/50 composition). This detailed analysis firstly explains the two spectral features at 62–70 eV (d-series) and 100–150 eV (s-series) for the four LMO samples. Secondly, it indicates one interesting insight: the observed spectral features are the effect of the material oxidation and atomic physics dynamics. In fact, there are differences that cannot be explained otherwise if one erroneously assumes that the plasma erases and thermalizes the entire ion signature, as in LIBS. Further, the + 2 (4 s shell) is not apparently fully neutralized upon recombination. Again, a half-full shell (here + 0.9 as given in Table 1) is not very receptive for electron recombination. Additional free electrons would occupy “unsaturated” shells in other ions in the plasma.

## Conclusions

This explorative study investigated the signal pattern in LIXS and put its radiative recombination component in relation to stoichiometric characteristics of the irradiated samples. It is shown that the spectra change, even for a given observation height, as a function of the oxidation of the sample. The observation height is shown to be important. In fact, the expansion of the plasma brings the optimum for the emission in different stages. This study shows a strong link to the fundamental quantum mechanics laws in the definition of the LIXS spectral structure. The fundamental insight is that the material stoichiometry selects the availability of unoccupied shells, which are mapped by the radiative recombination spectrum. This opens a way to use rapid laser plasma spectroscopy for quality control of the spatially resolved characteristics of a battery material, and the potential to failure as a consequence of deviation from the nominal stoichiometry of the functional materials.

## Data Availability

Data will be made available upon request.
